# Expression of *Msx1* and *Dlx1* during Dumbo rat head development: Correlation with morphological features

**DOI:** 10.1590/S1415-47572009005000041

**Published:** 2009-05-01

**Authors:** Suhair Katerji, Nathalie Vanmuylder, Michal Svoboda, Marcel Rooze, Stéphane Louryan

**Affiliations:** 1 Laboratory of Anatomy and Embryology, Faculté de Médecine, Université Libre de Bruxelles, BrusselsBelgium; 2 Laboratory of Biological Chemistry, Faculté de Médecine, Université Libre de Bruxelles, BrusselsBelgium

**Keywords:** Dumbo rat, Msx1, Dlx1, face, embryo, development

## Abstract

The Dumbo rat possesses some characteristics that evoke several human syndromes, such as Treacher-Collins: shortness of the maxillary, zygomatic and mandibular bones, and low position of the ears. Knowing that many homeobox genes are candidates in craniofacial development, we investigated the involvement of the Msx1 and Dlx1 genes in the Dumbo phenotype with the aim of understanding their possible role in abnormal craniofacial morphogenesis and examining the possibility of using Dumbo rat as an experimental model for understanding abnormal craniofacial development. We studied the expression of these genes during craniofacial morphogenesis by RT-PCR method. We used Dumbo embryos at E12 and E14 and included the Wistar strain as a control. Semi-quantitative PCR analysis demonstrated that *Msx1* and *Dlx1* are expressed differently between Dumbo and Wistar rats, indicating that their low expression may underly the Dumbo phenotype.

The “congenitally malformed” Dumbo rats seem to be the product of domestic breeding of rats of Wistar origin, probably in the USA, a few decades ago. They evoke comparisons with some human malformation syndromes, such as the Treacher-Collins, DiGeorge, and Nager syndromes, because of micrognathia, low position of the ears, and hypoplasia of the zygomatic, maxillary and mandibular bones (Figure1). This strain may constitute an experimental model for understanding abnormal craniofacial development**.**

Preliminary morphological and morphometic analysis indicated that the considerable differences between the craniofacial structures of Dumbo and Wistar rats might be due to genetic mutations in the Dumbo rat that were undetectable by chromosome mapping. Furthermore, the embryonic skulls of Dumbo rats displayed a delay bone growth. For these reasons, we selected the embryonic ages E12 and E14 (initiation of the chondrogenesis, beginning of ossification) for the present study.

Analysis of the embryonic development of Dumbo rats shows quantitative defects in structures derived from the first pharyngeal arch. These developmental defects are represented by disturbances in chondrogenesis and osteogenesis pathways, suggesting the involvement of the *Msx1* and *Dlx1* genes.

To compare genetic expression in Dumbo rats with the normal Wistar strain, we used RT-PCR to estimate the expression of *Msx1* and *Dlx1*. As loading controls we used the housekeeping gene glyceraldehyde-3-phosphate dehydrogenase (GAPDH), which is expressed at a constant level in different tissues, cells or experimental treatments (de Jonge *et al.*, 2007). Since the use of multiple internal control genes has been recommended (Vandesompele *et al.*, 2002), we used the nerve growth factor (NGF) encoding gene as second reference gene. NGF appears to be ubiquitously expressed in some craniofacial primordia during mouse development (Louryan *et al.*, 1995).

For the analyses on Dumbo rats, nine embryos at stages E12 and E14 were obtained from three different mothers, respectively. We also collected the same number of embryos for Wistar rats. Total RNA was extracted from small amounts of head tissue (20-100 mg) using the *RNA NOWTC* method (Texagen), according to the manufacturer's directions. The RNA pellet was dissolved in 50 μL of DEPC-treated water and RNA concentration was determined by spectrophotometry at 260 nm/ 280 nm using a Nanodrop ND1000 apparatus (Isogen). cDNA was synthesized by the *Gene Amp RNA PCR kit* (Applied Bio systems) using the enzyme MultiScibeRT (50U/μL): 1-2 μg of total RNA were transcribed in 20 μL of final volume of manufacture's buffer enriched with 2.5 μM random hexamers, 1 mM of dNTP and 20 units of RNAse inhibitor. Samples were incubated first at 25 °C for 10 min, then at 37 °C for 120 min. The reaction was stopped by the adition of 20 μL of 0.1 M EDTA and 30 μL water. The samples were heated to 94 °C for 2 min before storing at -20 °C. PCR reactions were set up in 20 μL, using the GoTaq PCR kit (Promega) with “Green buffer” and a final concentration of 200 μM dNTP, and 10 nM of each primer with 0.5 units of GoTaq DNA polymerase. Thermocycling wasperformed in MyCycler (BioRad), starting with a denaturation for 2 min at 94 °C, followed by cycles of 10 s at 94 °C, 20 s at 55-60 °C (depending on pair of primer used), 1 min at 72 °C. If not indicated otherwise, 35 amplifications cycles were done. After amplification, electrophoresis of 10 μL of each PCR product was performed on a 2% agarose gel with 0.5 μg/mL ethidium bromide, fragment size was estimated from a using 1 kb DNA ladder (Promega). To control for contamination of samples with genomic DNA, all PCR amplifications were carried out in parallel with a negative control of reverse transcription, *i.e.* with RNA samples submitted to reverse transcription but without MultiScibe Reverse Transcriptase. Semiquantitative RT-PCR estimates were validated using a standard curve dilution series of Wistar rat cDNA. Densitometries of amplicon fluorescence intensity were performed using VilberLourmat Bio1D software.

The RT-PCR analyses revealed that the expression of the Msx1 sense (S) gene, the Msx1 antisense (AS) gene and of the Dlx1 gene in the craniofacial region of E12 and E14 embryos was markedly lower in Dumbo rats than in Wistar rats (Figure 2). A very large difference was observed for the Msx1sense (S) gene, which was almost undetectable in Dumbo rats. Using dilution curves of Wistar cDNA, we validated that in our conditions the fluorescence intensity of amplicons was directly related to the initial concentration of target DNA. Using dilutions curves, we estimated that the expression of the Msx1 sense (S) gene in the Dumbo rat was one hundred times lower than in the Wistar rat. The difference between Dumbo and Wistar rats is significant (p = 0.0008). Expression of the Msx1antisense gene and of the Dlx1 gene in the Dumbo rat were roughly threefold lower than in the Wistar rat. The differences between Dumbo and Wistar rats were significant (p = 0.0008).As expected, the two rat strains did not differ significantly in the expression of the control genes: for GAPDH gene (p = 1.00) and for NGF gene (p = 0.87).

During embryogenesis, cranial neural crest cells migrate into the presumptive mandibular, maxillary and zygomatic primordia, where they condense to form mesenchymal and precartilaginous blastemata before differentiating into osteoblasts. The osteoblasts synthesize bone matrix through intramembranous ossification (Couly *et al.*, 1993; Hall and Miyake, 2000), whereas the ossicles of ear, derived from Meckel's and Reichert's cartilage form through endochondral ossification. Normal development requires mechanisms to ensure that bone morphology and growth are matched to those of the developing skull (Morriss-Kay, 1993).

The generation of different cell types from cranial neural crest (CNC) is regulated by genetic control, which is beginning to be elucidated, as a large number of candidate regulatory genes identified and mutations in these genes are being made. Some of these genes are homeobox genes. They are expressed in the early phases of development in a spatially and temporally restricted manner and have been implicated in the specification of particular domains of the head. Homeobox genes are a conserved ubiquitous superfamily of transcription factors found in all eukaryotes, with analogs in prokaryotes. In eukaryotic organisms, these genes generally regulate axis determination, segmental patterning, and tissue identity during development. The protein product of a homeobox gene contains a highly conserved homeodomain at the carboxyl end that includes a DNA binding helix-turn-helix motif. Homeobox proteins also contain a variable region composed of one or more domains involved in protein binding specificity and regulation (Qian *et al.*, 1989; Kissinger *et al.*, 1990)

The Msx and Dlx homeobox gene families are expressed in the pharyngeal arches, giving rise to craniofacial structures. The mandible, maxilla, zygoma and ear are derived from the first pharyngeal arch, which receives neural crest cells from the midbrain, namely the mesencephalon, and rhombomeres r1 and r2 (Tan and Morriss-Kay, 1985). The expression of the Msx and Dlx gene families in the cranial neural crest cells emigrating from the central nervous system continues in the craniofacial regions.

The Msx1 gene is strongly expressed in CNC (cranial neural crest) cells and plays a critical role in regulating epithelial-mesenchymal transitions during morphogenesis (Robert *et al.*, 1989). Expression of *Msx1* in the cranial neural crest continues during cell migration and colonization of the pharyngeal arches (Mackenzie *et al.*, 1992). In the central nervous system, the expression of *Msx1* is essential in the hindbrain and the rhombomeres. Dorsolateral expression of *Msx1* continues in the brain during neurulation and becomes more lateral (Bendall and Abate-Shen, 2000).

*Dlx1* is a member of the distal-less homeobox gene family. It is likely to be responsible for programming developmental events along the proximodistal and mediolateral dimensions of the pharyngeal arches (Qiu *et al.*, 1995). Dlx genes establish intra-arch identity (Depew *et al.*, 2005). Since the CNC contributing to the maxillary and mandibular components of the first arch is derived from the posterior midbrain and rhombomeres 1 and 2 (Osumi-Yamashita *et al.*, 1994), candidate regulators of the Dlx genes must be expressed in this neuroepithelium.

Our findings may help to explain the delayed chondrogenesis and the late osseous growth of these regions in Dumbo rats in comparison with Wistar rats. Blin-Wakkach *et al.*, (2001) demonstrated the existence of endogenous Msx1 antisense RNA (Msx1-AS RNA) in differentiated dental and craniofacial tissues of mice, rats, and humans. They also showed that this AS RNA can block Msx1 protein expression and that it exhibits a reverse temporospatial distribution pattern with Msx1 protein both *in vivo* and *in vitro*.

*Msx1-S* is expressed strongly in the proliferative progenitor cells of dental mesenchyme and bone, and it is down-regulated in terminally differentiated tissues (Robert *et al.*, 1989; Mackenzie *et al.*, 1991; Houzelstein *et al.*, 1997). By contrast, an inverse distribution of the Msx1-AS RNA was shown by Blin-Wakkach *et al.* (2001). These authors showed that when the AS transcript is more abundant, Msx1 protein is undetectable, and conversely, overexpression of the sense RNA results in production of Msx1 protein. They next demonstrated that the balance between the levels of the two Msx1 RNAs (sense/antisense) is related to the expression of Msx1 protein and that this ratio is very important in the control of terminal differentiation of the skeleton. They also demonstrated that the Msx1-AS RNA is involved in a cross talk between the Msx-Dlx pathways. Forest-Potts and Sadler (1997) highlighted that antisense attenuation of Msx1 during early stages of neurulation led to hypoplasia of the maxillary and mandibular bones, and to abnormalities in the neural tube. When cultured mouse embryos were injected with Msx1-AS oligodeoxynucleotides, expression of Msx1 protein was disrupted and craniofacial abnormalities ensued. Msx1 was shown to down-regulate the master gene of osteoblastic determination, *Cbfa1*, a strongly indication that the ratio between Msx1-S and Msx1-AS RNA is a key factor in cell differentiation and phenotypic expression in mineralized tissues (Blin-Wakkach *et al.*, 2001). Because the expression patterns of the Msx genes are closely related to the development of neural crest cells in several species, the failure of early craniofacial development could be due to aberrant CNC cells induction or migration. Han *et al.*, (2007) reported that the Msx1 gene is specifically required for osteogenesis in the cranial neural crest lineage. They showed that differentiation of the mesenchyme and establishment of certain craniofacial structures was defective in *Msx1*^*-/-*^mice. They also showed that the failure of CNC-derived mesenchymal cells to express *Runx2* and Osterix in the absence of Msx1 may prevent osteogenic differentiation*. Runx2* is an essential transcription factor controlling osteoblast differentiation. Null mutation of *Runx2* leads to a complete lack of ossification in both neural crest and mesoderm derived bones (Komori *et al.*, 1997).

Targeted null mutation of *Msx1* results in multiple craniofacial abnormalities involving a defect in mandibular bone development**.** In humans, mutations in the Msx1 gene have been implicated in tooth agenesis (Padanilam *et al.*, 1992; Hu *et al.*, 1998) and cleft palate (Van Den Boogaard *et al.*, 2000), and the phenotype was proposed to be related to a dose effect of Msx1 protein (Hu *et al.*, 1998). Interestingly, *Msx1* down-regulation is associated with the terminal differentiation of several cell types, such as cartilage (Mackenzie *et al.*, 1991; Coelho *et al.*, 1993; Mina *et al.*, 1995) and muscle (Houzelstein *et al.*, 1999).

Our data indicate that expression of the Dlx1 gene at the E12 and E14 stages during craniofacial development is weaker in the Dumbo rat than in Wistar rat. The reduced expression of the Dlx1 gene in Dumbo rats might be implicated in the malformed genesis of the head in these rats. Depew *et al.* (2002, 2005) showed that *Dlx*-mutant mice exhibit severe craniofacial deformities, including cleft palate, and dysmorphic middle ear and jawbones. *Dlx*-mutant mice show delayed ossification of dermal bones (Merlo *et al.*, 2000) resembling the defects caused by inactivation of one copy of *Cbfa1* (Otto *et al.*, 1997). It seems that both *Msx1* and *Dlx1* have a direct or indirect relation with *Cbfa1.*

Kim *et al.*, (1998) showed that *Fgfr2* expression was reduced in the craniofacial structures of *Msx1*^*-/-*^ mouse embryos. There is evidence that FGF signalling is involved in calvarial development. In calvarial culture, FGF4 accelerates ossification. FGF2 can rescue the compromised osteogenitor proliferation of *Tgfr2* conditional knockout mice (Sasaki *et al.*, 2006). Robel *et al.*, (1995) showed that FGF2 increased Dlx1 expression and that this effect was gene-specific, dose-dependent, and temporally regulated, with larger effects at earlier stages of development. This interaction between FGF2 and Dlx1 may be important for the regulation of the antero-posterior pattern in craniofacial development. Zhang *et al.*, (1997) showed that some of the defects in Msx1^-/-^ mice may be aggravated or rescued by controlling certain Dlx genes. The essential condition for this regulation to occur is that the two genes be expressed in the same cells at the same time.

In conclusion, we found that the Msx1 and Dlx1 genes are expressed differently during head development of Dumbo and Wistar rats, with a reduction of expression in the Dumbo strain. This suggests that the Dumbo rat could be a suitable experimental model for understanding abnormal craniofacial development. This rat reflects the relation between some homeobox genes and the craniofacial abnormalities. The search for other concomitant events related to craniofacial abnormalities will be necessary, such as studying apoptosis and the involvement of other genes in the Dumbo phenotype. Confirmation of our findings alsom requires studying the expression of the implicated genes by *in situ* hybridization and by investigating the expression of Msx1 protein by Western blot analysis.

**Figure 1:**
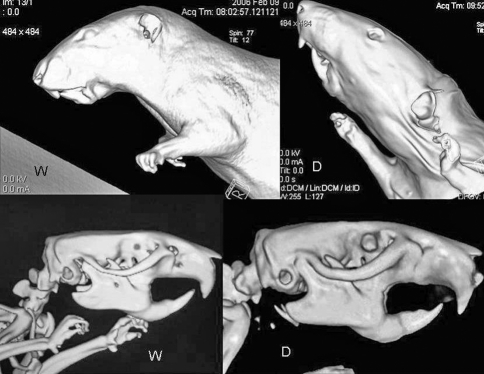
CT scanning 3D reconstruction of Wistar (W) and Dumbo (D) adult rats. Upper panel: cutaneous reconstruction; lower panel: skeletal reconstruction. Note the low-situated ears, short zygomatic bone, thin tympanic ring, and short snout and mandible in the Dumbo strain.

**Figure 2:**
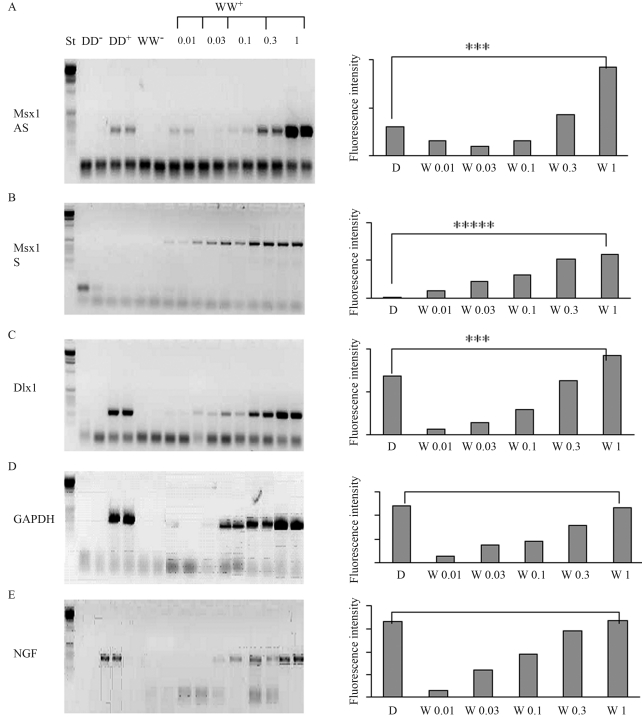
Comparison by RT-PCR analysis of the expression of the Msx1-AS gene, the Msx1-S gene, and the Dlx1 gene between Dumbo and Wistar rats during craniofacial morphogenesis. PCR products were separated by electrophoresis on 2% agarose gel and stained with ethidium bromide. DD^-^: negative control of Dumbo cDNA. DD^+^: positive control of Dumbo cDNA. WW^-^: negative control of Wistar cDNA. WW^+^: positive control of Wistar cDNA with a dilution series of Wistar cDNA :from 0.01-0.03-0.1-0.3-1.0 The expression of the Msx1-AS gene and the Msx1-S gene were markedly lower in Dumbo rats compared to the Wistar strain (p = 0.0008). Expression of the NGF encoding gene was identical in both strains (= 0.87).
